# Island-like Perovskite Photoelectric Synaptic Transistor with ZnO Channel Layer Deposited by Low-Temperature Atomic Layer Deposition

**DOI:** 10.3390/ma18122879

**Published:** 2025-06-18

**Authors:** Jiahui Liu, Yuliang Ye, Zunxian Yang

**Affiliations:** 1 College of Information Science and Engineering, Huaqiao University, Xiamen 361021, China; 2 National & Local United Engineering Laboratory of Flat Panel Display Technology, Fuzhou University, Fuzhou 350108, China

**Keywords:** synaptic hybrid phototransistors, island-like perovskite film, metal oxide, atomic layer deposition, quiescent current

## Abstract

Artificial photoelectric synapses exhibit great potential for overcoming the Von Neumann bottleneck in computational systems. All-inorganic halide perovskites hold considerable promise in photoelectric synapses due to their superior photon-harvesting efficiency. In this study, a novel wavy-structured CsPbBr_3_/ZnO hybrid film was realized by depositing zinc oxide (ZnO) onto island-like CsPbBr_3_ film via atomic layer deposition (ALD) at 70 °C. Due to the capability of ALD to grow high-quality films over small surface areas, dense and thin ZnO film filled the gaps between the island-shaped CsPbBr_3_ grains, thereby enabling reduced light-absorption losses and efficient charge transport between the CsPbBr_3_ light absorber and the ZnO electron-transport layer. This ZnO/island-like CsPbBr_3_ hybrid synaptic transistor could operate at a drain-source voltage of 1.0 V and a gate-source voltage of 0 V triggered by green light (500 nm) pulses with low light intensities of 0.035 mW/cm^2^. The device exhibited a quiescent current of ~0.5 nA. Notably, after patterning, it achieved a significantly reduced off-state current of 10^−11^ A and decreased the quiescent current to 0.02 nA. In addition, this transistor was able to mimic fundamental synaptic behaviors, including excitatory postsynaptic currents (EPSCs), paired-pulse facilitation (PPF), short-term to long-term plasticity (STP to LTP) transitions, and learning-experience behaviors. This straightforward strategy demonstrates the possibility of utilizing neuromorphic synaptic device applications under low voltage and weak light conditions.

## 1. Introduction

Von Neumann computers suffer from the bottleneck problem of signal delay and high energy consumption in information processing due to the physical separation of the processing units and memory [1,2,3]. Emerging neuromorphic computing represents a promising advance in overcoming these limitations [4,5]. In order to realize neuromorphic computing in hardware, the study of artificial synaptic devices is a necessary step. Among artificial synaptic devices, photoelectric synaptic transistors, which have wireless communication and ultra-fast signal transmission, avoid the trade-off between bandwidth and connection density [2,5,6]. Therefore, photoelectric synaptic transistors have received more and more attention in recent years, with continuous research and development in this field. So far, various materials have been explored for artificial photoelectric synapses. In particular, inorganic metal halide perovskites have been widely used as promising light-absorbers in neuromorphic photoelectric devices due to their exceptional light absorption, tunable band gap, and easy fabrication [7,8,9,10,11]. However, their low carrier transport and the presence of ion migration in perovskite films cause high power consumption, large leakage currents, device instability, etc. [10,12,13,14]. An effective strategy to improve the carrier transport of perovskite-based photoelectric synapses is to construct type II heterostructures by combining perovskites with carrier transport layers. Up to now, various carrier transport materials have been utilized, including organic semiconductors [15,16,17,18], low-dimensional materials [19,20,21], metal oxide [22,23,24,25], etc. ZnO, as an excellent electron-transport material, has a lot of advantages, such as high structure stability and large-scale deposition compatibility. Currently reported studies have successfully employed the magnetron sputtering technique to fabricate ZnO/perovskite hybrid layers for photoelectric synaptic devices [22,26,27]. Despite the progress made, these perovskite-based devices usually suffer high quiescent currents, causing extra energy consumption during device operation. Another issue is that sputtered ZnO films often exhibit poor uniformity with intrinsic defects, particularly on rough perovskite surfaces, thus hindering effective charge extraction. To improve the EPSC response of a synaptic device, it is necessary to fabricate a high-quality perovskite hybrid film. Alternatively, ALD is a good choice for depositing ZnO to form the ZnO/perovskite hybrid layer. In photovoltaic and light-emitting devices, ZnO films deposited by ALD offer many advantages, including exceptional uniformity, high compactness, and low-temperature processing capability (<200 °C), etc. [28,29]. It is noteworthy that our previous study demonstrated that perovskite films with island-like morphology could mitigate the impact of ion migration on the gate voltages and suppress the state-off currents of devices [18]. This is beneficial for reducing the quiescent currents in devices when no input signal is applied. Since ALD relies on gas-phase precursors, this technique enables infiltration between grains of perovskite polycrystalline film, which facilitates the formation of well-defined perovskite/ZnO interfacial contacts.

In this study, a novel wavy-structured CsPbBr_3_/ZnO hybrid film was realized by depositing dense and thin ZnO film via low-temperature ALD on island-like CsPbBr_3_ perovskite film. Considering the temperature sensitivity of perovskites, the influence of the ALD deposition temperature on the island-like CsPbBr_3_ films was investigated. To mitigate the impact of perovskite ion migration on the gate electric field modulation, the spin-coating rotation speed for CsPbBr_3_ film fabrication was optimized. Due to the capability of ALD to grow high-quality films over small surface areas, dense ZnO films were deposited on the substrate, filling the gaps between the island-shaped CsPbBr_3_ grains. The ZnO/island-like CsPbBr_3_ hybrid synaptic transistors were able to operate at a bias voltage of 1.0 V under green light (500 nm) pulses with light intensities of as low as 0.035 mW/cm^2^. The island-like CsPbBr_3_/ALD-ZnO hybrid transistor exhibited a quiescent current of ~0.5 nA. Notably, the patterned device achieved a significantly reduced off-state current of 10^−11^ A and lowered the quiescent current to 0.02 nA. This resulting transistor could emulate fundamental synaptic behaviors, including EPSC, PPF, STP-to-LTP transitions, and learning-experience behaviors. These findings highlight the promising potential of such ZnO/island-like CsPbBr_3_ heterostructures for neuromorphic synaptic device applications.

## 2. Materials and Methods

### 2.1. Precursor and Hybrid Transistor Fabrication

The 0.165 M CsPbBr_3_ precursor was acquired via the dissolution of PbBr_2_ (99.999%) and CsBr (99.999%) at a molar ratio of 1:1 in dimethyl sulfoxide (DMSO, 99.9%). The CsPbBr_3_ (preheated at 100 °C) precursor was utilized for spin-coating at 6000 rpm for 60 s under nitrogen atmospheres on the cleaned and hydrophilic Si/SiO_2_ substrate in order to form an island-like CsPbBr_3_ film. The resulting CsPbBr_3_ film was then transferred to a pump chamber for removing the residual solvent, followed by annealing at 100 °C for 5 min. For the ALD process, diethylzinc (DEZ) and deionized water (H_2_O) were used as precursors to deposit ZnO, and the reactor was kept at 70 °C. The equipment started to run according to the designed program: the DEZ remained at a pulse duration of 0.02 s with a purging time of 30 s, and the H_2_O remained at a pulse duration of 0.02 s with a purging time of 40 s at a chamber pressure of 20 Pa. The ~30 nm ZnO layer was deposited with 280 cycles. Finally, 100 nm-thick aluminum electrodes with channel lengths of 80 μm and widths of 200 μm were formed by the shadow mask thermal evaporation method to fabricate the hybrid phototransistors.

### 2.2. Characterization and Measurements

The morphologies of the films were obtained by means of atomic force microscopy (AFM, Bruker, Birrica, MA, USA) and a fluorescence microscope (Olympus, BX51M, Tokyo, Japan). The photoelectric properties of the films were analyzed by steady-state photoluminescence (PL, obtained by an F4600 spectrometer) and the UV-Vis absorption spectra (obtained by a UV/vis/NIR spectrophotometer, Hitachi, Tokyo, Japan). The material structure was investigated via the X-ray diffraction (XRD) spectra of the films using a X-ray diffractometer with Cu Ka radiation (l = 1.5418 Å; Philips, Amsterdam, The Netherlands). The surface potential of the films was measured by means of the Kelvin probe force microscopy (KPFM) under 500 nm illumination. The electrical measurements of the devices were conducted within an ambient environment at a room temperature by utilizing the Keithley 4200-SCS (Tektronix, Beaverton, OR, USA). The data results in our manuscript were not averaged.

## 3. Results and Discussion

### 3.1. Fabrication of Island-like CsPbBr_3_/ZnO Hybrid Synaptic Transistors

**Figure 1:**
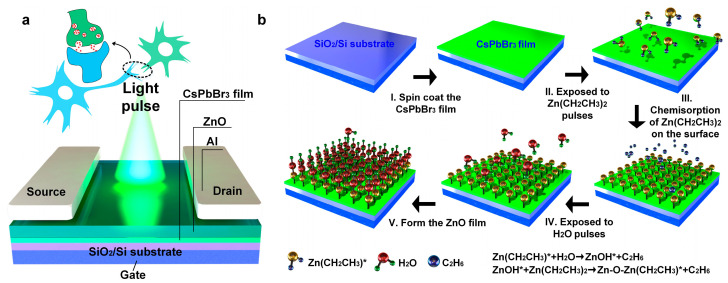
(**a**) Schematic diagram of the device structure of a photoelectric synaptic transistor based on CsPbBr_3_/ZnO hybrid film. (**b**) Schematic diagram of the preparation process of the CsPbBr_3_/ZnO hybrid film.

In the human brain, synapses serve as connections between neurons. Presynaptic neurons respond to stimuli and then release neurotransmitters that bind to receptors in postsynaptic neurons [2,3]. Similar to synapses in the human visual system, photoelectric synaptic transistors can convert received optical signals into electrical signals. Figure 1a shows the schematic structure of the island-like CsPbBr_3_/ZnO hybrid synaptic transistor. The island-structured CsPbBr_3_ film on the SiO_2_/Si substrate acts as the light-absorbing layer, while the ZnO layer deposited in the gaps between the CsPbBr_3_ islands acts as the charge transport layer. The fabrication process flow of the CsPbBr_3_/ZnO hybrid film is shown in Figure 1b. Initially, the CsPbBr_3_ film was fabricated on the SiO_2_/Si substrate via the process of spin-coating. Then, ZnO was deposited on the surface of the CsPbBr_3_ film by ALD. The ALD process used DEZ as the metal precursor and H_2_O as the oxidant precursor. Considering the sensitivity of perovskite materials to factors such as moisture and oxygen, the DEZ precursor was first introduced to adhere to the surfaces of the CsPbBr_3_ and the substrate. The excess DEZ was then purged using a nitrogen gas flow. The H_2_O precursor was then introduced to chemically react with the DEZ on the surfaces to form ZnO. By controlling the number of DEZ/H_2_O precursor cycles, the desired thicknesses of the ZnO films were achieved.

### 3.2. Effect of ALD-Grown ZnO Process on the CsPbBr_3_/ZnO Hybrid Film

**Figure 2:**
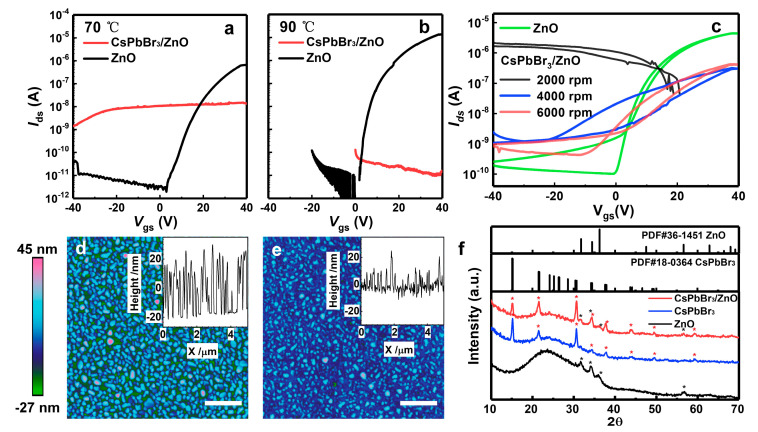
Transfer curves of the ZnO transistor and the CsPbBr_3_/ZnO transistor with the ZnO layer deposited via ALD at (**a**) 70 °C and (**b**) 90 °C, respectively. (**c**) Transfer curves of the CsPbBr_3_/ZnO hybrid transistor based on CsPbBr_3_ films spin-coated at different speeds (2000–6000 rpm). AFM image of (**d**) the CsPbBr_3_ film and (**e**) the CsPbBr_3_/ZnO hybrid film (scale bar: 1 μm; inset: height and line-scan profiles). (**f**) XRD patterns of CsPbBr_3_, ZnO, and CsPbBr_3_/ZnO film.

Substrate temperature is a critical factor affecting the quality of ALD-grown CsPbBr_3_/ZnO hybrid films. On one hand, the quality of ZnO films determines the electrical performance of ZnO-based thin-film transistors. On the other hand, perovskite materials are highly temperature-sensitive. Therefore, the effects of substrate temperatures (70 °C and 90 °C) on the electrical properties of CsPbBr_3_/ZnO hybrid transistors were investigated. The electrical characterization shows that the higher ALD substrate temperatures achieved better electrical performance of the ZnO transistor, with the on-state current increasing from ~10^−6^ A to ~10^−5^ A (Figure 2a,b). In contrast, the on-state current of the CsPbBr_3_/ZnO hybrid transistor decreased drastically to ~10^−11^ A, indicating a high-resistance state. In comparison, the hybrid transistor fabricated at 70 °C exhibited an on-state current of ~10^−8^ A. Combined with the morphological analysis, the performance degradation at 90 °C is possibly attributed to the coalescence and growth of CsPbBr_3_ grains, which blocked the lateral conduction channels of the ZnO film. When the ALD substrate temperature was set at 70 °C, the AFM images showed a uniform particle distribution of the CsPbBr_3_/ZnO hybrid film (Figure S1a). However, when the substrate temperature was increased to 90 °C, partial grain coalescence occurred in the CsPbBr_3_ film during the ALD processing, resulting in larger grain sizes and degraded film uniformity (Figure S1b). This obstruction significantly reduced the carrier transport paths along the lateral direction, resulting in the deterioration of the electrical performance. Therefore, to achieve the optimal electrical performance in the hybrid transistor, the ALD substrate temperature was selected as 70 °C for the fabrication of the CsPbBr_3_/ZnO hybrid film. Further investigation focused on the influence of CsPbBr_3_ films prepared at different spin-coating speeds on the electrical properties of the device. As shown in Figure 2c, the transfer characteristic curves of the CsPbBr_3_/ZnO transistors based on the CsPbBr_3_ films were spin-coated at different speeds (2000–6000 rpm). At a spin-coating speed of 2000 rpm, the hybrid devices exhibited no transistor characteristics. Transistor transfer curves appeared when the spin speed exceeded 4000 rpm. In this range, the off-state current of the device was approximately 10^−9^ A. As the spin speed increased from 4000 to 6000 rpm, the threshold voltage decreased from −20 V to −10 V, the off-state current decreased to ~10^−10^ A, and the hysteresis window narrowed. Previous studies have reported that ion migration in perovskites can induce electric field shielding under gate bias. The insertion of a CsPbBr_3_ layer between a ZnO layer and a SiO_2_ dielectric has been shown to weaken the gate bias modulation of the ZnO channel conductivity and exacerbate device hysteresis. Figure 2d shows that the CsPbBr_3_ film spin-coated at 6000 rpm exhibits an island-like distribution. After the ALD deposition of the ZnO film, it was observed that dense and thin ZnO film filled the gaps between the island-shaped CsPbBr_3_ grains, forming a wave-shaped CsPbBr_3_/ZnO hybrid film (Figure 2e). To investigate the effect of the ALD process on the CsPbBr_3_ film, the material structures of the ZnO, CsPbBr_3_, and CsPbBr_3_/ZnO hybrid film were tested by XRD measurement. The XRD patterns in Figure 2f confirm that the ZnO deposited at 70 °C has a well-defined wurtzite structure (PDF36-1451), while the CsPbBr_3_ has adopted an orthorhombic phase (PDF18-0364). The CsPbBr_3_/ZnO hybrid film shows peaks corresponding to both the ZnO and CsPbBr_3_, with no additional phases detected. This indicates that the ALD deposition of the ZnO on the CsPbBr_3_ did not induce phase transformation in the perovskite under the chosen conditions.

### 3.3. Optoelectronic Characterization of the CsPbBr_3_/ZnO Hybrid Film

**Figure 3:**
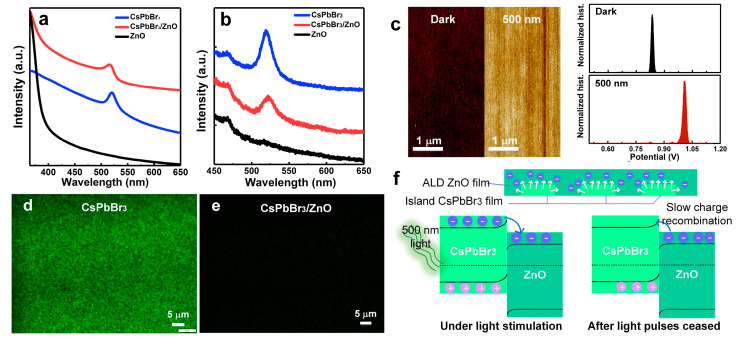
(**a**) UV-Vis and (**b**) PL spectra of CsPbBr_3_, ZnO, and CsPbBr_3_/ZnO films, respectively. (**c**) Surface potential of the CsPbBr_3_/ZnO film under 500 nm light and dark. Microscope images of (**d**) CsPbBr_3_ and (**e**) CsPbBr_3_/ZnO films under blue light. (**f**) Energy-band diagram of hybrid device under 500 nm light illumination and after light pulses ceased.

As shown in Figure 3a, the CsPbBr_3_/ZnO hybrid film exhibited significantly enhanced light absorption in the 365–500 nm wavelength range compared to pure CsPbBr_3_ and ZnO. Figure 3b shows that the PL emission maximum of the CsPbBr_3_ was significantly quenched at about 520 nm, suggesting the photogenerated carriers exhibited an efficient separation at the CsPbBr_3_/ZnO interface. Furthermore, an obvious increase in the surface potential of the CsPbBr_3_/ZnO hybrid film was observed under green light compared with the condition in the dark, confirming the transfer of photogenerated carriers from the CsPbBr_3_ to the ZnO (Figure 3c). Figure 3d,e presents fluorescence microscopy images of the CsPbBr_3_ and CsPbBr_3_/ZnO films (spin-coated at 6000 rpm) under blue light. The hybrid film exhibits fluorescence quenching compared to the pure CsPbBr_3_. As shown in the XRD results (Figure 2f), the presence of the CsPbBr_3_ peaks in the hybrid film confirms that the fluorescence quenching was due to the charge separation at the CsPbBr_3_/ZnO heterojunction rather than phase degradation. To attain a sufficient photocurrent, devices with thermally evaporated aluminum (Al), silver (Ag), and gold (Au) electrodes were studied (Figures S2 and S3). Figure S2a–f exhibit that the deposited ZnO film has failed to suppress the migration of silver and bromide ions and their subsequent redox reactions. In contrast, the CsPbBr_3_/ZnO film using Al electrodes remains stable (Figure S2g,h). Additionally, the use of gold electrodes in CsPbBr_3_/ZnO hybrid transistors results in poor electrical performance, primarily due to energy-level misalignment at the interface (Figure S3). Consequently, Al is selected as the preferred electrode material for subsequent CsPbBr_3_/ZnO hybrid transistors. The energy band diagram in Figure S4a represents that the conduction band minimum and valence band maximum of CsPbBr_3_ were −5.94 eV and −7.65 eV while those of ZnO were −3.55 eV and −4.3 eV. Since the CsPbBr_3_ and ZnO formed a type II heterojunction, the separation of the photogenerated carriers occurred rapidly at the CsPbBr_3_/ZnO interface, owing to the built-in potential (Figure 3f). Electrons transferred to the ZnO layer, increasing the channel conductivity, while holes remained trapped in the CsPbBr_3_ (Figure 3f). This suppressed the electron–hole recombination and prolonged the lifetime of the electrons in the ZnO. Even after the light pulses ceased, the trapped holes in the CsPbBr_3_ delayed the electron recombination in the ZnO, resulting in a slow current-decay behavior analogous to EPSC decay in biological synapses. Moreover, the island-like CsPbBr_3_ film enlarged the interface area, which was beneficial for the fast separation of photoexcited carriers. Figure S4b displays an obvious voltage shift toward the negative direction of the transfer characteristic curves for the CsPbBr_3_/ZnO hybrid transistor under 500 nm light illumination. It can be seen that the ZnO transistor had a poor photosensitive performance with light illumination. This result indicates the effective extraction of photoexcited electrons into the ZnO layer from the island-like CsPbBr_3_ film.

### 3.4. Behavior of the CsPbBr_3_/ZnO Hybrid Photoelectric Synaptic Transistor

**Figure 4:**
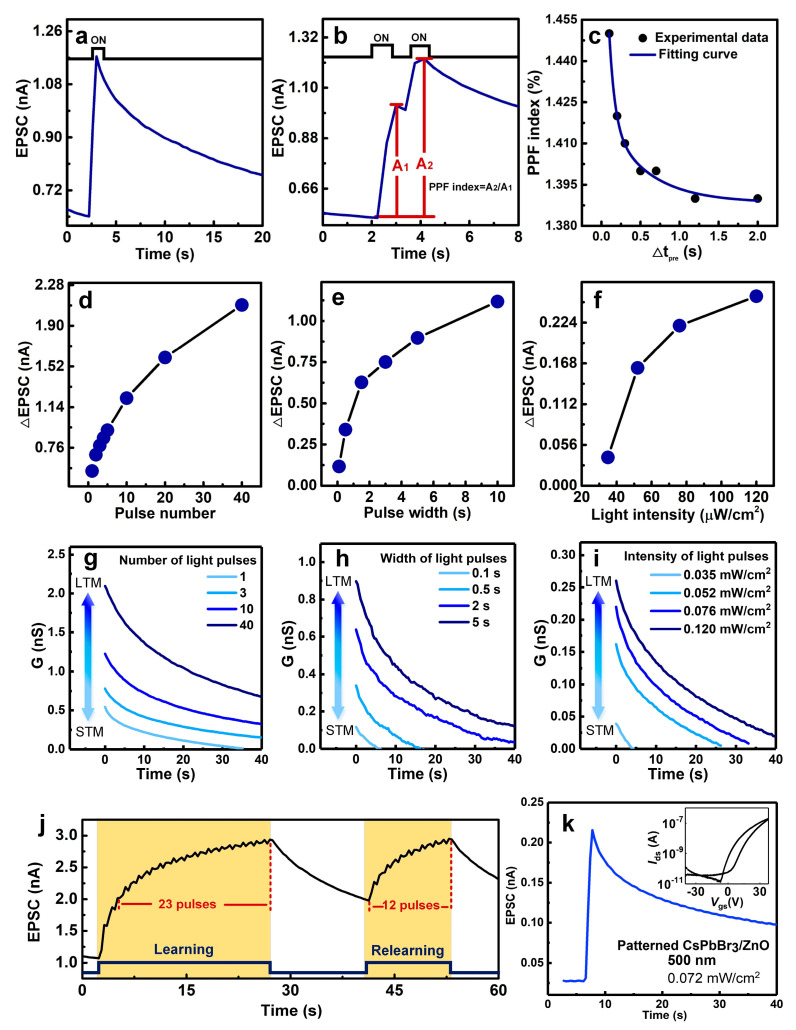
(**a**) EPSC behavior of the CsPbBr_3_/ZnO hybrid transistor triggered by a light pulse. (**b**) PPT behavior of the hybrid phototransistor triggered by two successive light spikes. (**c**) PPF index as a function of the time interval between two successive light pulses. ΔEPSC as a function of the (**d**) number, (**e**) width, and (**f**) intensity of the light pulse. Emulation of STM and LTM behaviors by varying the (**g**) number, (**h**) width, and (**i**) intensity of the light pulse. (**j**) Learning experience of the hybrid transistor. (**k**) EPSC of the patterned CsPbBr_3_/ZnO hybrid transistor (inset: transfer characteristic curve of the patterned device).

To verify the behavior of the CsPbBr_3_/ZnO synaptic transistors, the drain-source voltage and gate bias were set to 1 V and 0 V, respectively. Figure 4a depicts the typical EPSC behavior of the hybrid transistor under a 500 nm monochromatic light pulse (pulse width: 0.5 s and light intensity: 0.25 mW/cm^2^). The EPSC demonstrated a pronounced increase in response to the light pulse and then exhibited a slow decay after removing of the light pulse. The extracted ΔEPSC (defined as the amplitude of the EPSC) was ~0.67 nA, and the quiescent current was as low as ~0.5 nA. As illustrated in Figure 4b, the PPF was triggered by two consecutive light pulses (pulse width of 0.5 s, time interval Δ*t*_pre_ of 0.5 s) and the second ΔEPSC (*A*_2_) exceeded that of the first (*A*_1_). Due to the presence of a barrier between the island-like CsPbBr_3_ and ZnO, residual electrons from the first pulse accumulated in the ZnO channel. This resulted in a stronger EPSC when the second pulse was applied, mimicking the PPF behavior observed in biological synapses. The PPF index was calculated as [10]:

(1) $$\mathrm{PPF}=\left(A_{2}-A_{1}\right)/A_{1}\times 100\%$$

where *A*_1_ and *A*_2_ represent the ΔEPSC values of the first and second pulses, respectively. Figure 4c illustrates the PPF index decreasing following an exponential decay as Δ*t*_pre_ increases. Additionally, the EPSC of the device could be adjusted via the parameters of the light pulses, such as the number, duration, and intensity (Figure 4d–f). In Figure 4d, it can be observed that when the number of light pulses was less than 10, there was a significant increase in the ΔEPSC. However, as the number of pulses continued to increase, the rate of the enhancement slowed down. This phenomenon may be related to the saturation of the light absorption in the material [14]. Similar trends in ΔEPSC were also observed with increasing light pulse width and light intensity (shown in Figure 4e,f). As shown in Figure S5, the α was obtained at lower than 1 from the fitted line following the power-law, suggesting the obvious photogating effects for the enhancement of the photocurrent [30]. Synaptic plasticity is divided into STP and LTP based on its duration [31]. Similarly, memory retention is broadly divided into short-term memory (STM) and long-term memory (LTM), with LTM resulting from the transition of STP to LTP [31]. Repeated stimulation can convert STP into LTP, which is critical for learning and memory. For the synaptic device, the channel conductance (G) can be regarded as an analogue for memory retention. Figure 4g–i depict the effects of the properties of light pulses on G based on the level of memory. As shown in Figure 4g, G decays more slowly as the number of applied light pulses applied increases (1, 3, 10, and 40 pulses), reflecting the prolonged retention of memory in the human brain. Similarly, increasing the pulse duration (Figure 4h) and light intensity (Figure 4i) facilitated the transition from STM to LTM. These results demonstrate the device’s ability to emulate synaptic learning rules and forgetting behaviors, exhibiting the potential to be of use in the future of neuromorphic computing. Figure 4j illustrates the hybrid photoelectric synaptic transistor that mimicked the human repetitive learning–forgetting process. The number of light pulses is analogous to the number of learning repetitions. During the initial learning phase, 25 light pulses (pulse width of 0.5 s, Δ*t*_pre_ of 0.5 s) were applied to trigger the CsPbBr_3_/ZnO hybrid channel, which significantly increased the EPSC amplitude, corresponding to improved memory retention after 25 learning cycles. Over time, the EPSC spontaneously decayed to a specific level, simulating the forgetting process. In the second learning phase, only 12 light pulses, fewer than the 23 pulses required in the first phase, were needed to restore the EPSC to its initial level. This mirrors the human brain’s ability to relearn forgotten information with fewer repetitions. Additionally, the low quiescent current in devices when there is no input signal is essential for synaptic devices. Figure 4k shows the transfer characteristic curves of a patterned CsPbBr_3_/ZnO hybrid transistor. Using a probe, the CsPbBr_3_/ZnO film was mechanically patterned with dimensions of ~500 μm × 500 μm. This device exhibited a significant increase in the on/off current ratio of 10^4^ accompanied by a narrower hysteresis window. Notably, it can be observed that there is a reduced off-state current of the patterned transistor (down to ~10^−11^ A) compared with that of the non-patterned device (~10^−9^ A). The lower off-state current is beneficial for optimizing the quiescent current. The EPSC of this patterned CsPbBr_3_/ZnO hybrid transistor under a 500 nm light pulse has a low quiescent current of 0.025 nA and exhibits a markedly slower decay than that of the non-patterned device. This difference likely arose because the patterning of the composite film suppresses fringe effects [32], highlighting the necessity of patterning hybrid films for advanced synaptic functionality. The comparison in Table S1 represents that the quiescent current for island-like CsPbBr_3_/ALD ZnO hybrid transistors, especially for patterned devices, is lower than those of most other photoelectric synaptic devices in previous studies. However, the EPSC amplitude shows no significant increase. This may require future studies to focus on interface optimization and performance enhancement of the zinc oxide transport layer, aiming to improve photogenerated carrier extraction.

## 4. Conclusions

To summarize, a hybrid photoelectric synaptic phototransistor based on island-like CsPbBr_3_ film/ZnO is proposed. The dense ZnO film was fabricated by ALD at a low temperature of 70 °C, filling the gaps between the island-shaped CsPbBr_3_ grains. To reduce the hysteresis of the devices, the spin-coating rotation speed for the CsPbBr_3_ film fabrication was optimized at 6000 rpm. The ZnO/island-like CsPbBr_3_ hybrid synaptic transistors were able to operate at a bias voltage of 1.0 V under green light (500 nm) pulses with light intensities of as low as 0.035 mW/cm^2^. The device exhibited a quiescent current of ~0.5 nA. Notably, after patterning, it achieved a significantly reduced off-state current of 10^−11^ A and decreased the quiescent current to 0.02 nA. This resulting transistor could emulate fundamental synaptic behaviors, including EPSCs, PPF, STP to LTP transitions, and learning-experience behaviors. These characteristics made this island-like CsPbBr_3_ film/ZnO hybrid photoelectric transistor suitable for future artificial neuromorphic human vision.

## Data Availability

The original contributions presented in this study are included in the article. Further inquiries can be directed to the corresponding authors.

## Supplementary Materials

The following supporting information can be downloaded at: https://www.mdpi.com/article/10.3390/ma18122879/s1, Figure S1: AFM image of CsPbBr_3_/ZnO hybrid film with the ZnO film deposited via ALD at substrate temperature of (a) 70 °C and (b) 90 °C (scale bar: 1 μm). AFM image of the CsPbBr_3_ films spin-coated at (c) 2000 rpm and (d) 3000 rpm (scale bar: 1 μm); Figure S2: Microscopy images of CsPbBr_3_/ZnO hybrid Films with silver and Al electrodes under white light (a,c,e–g) and blue light (b,d,h): (a,b) Hybrid film with Ag electrodes. (c,d) Hybrid film with silver electrodes aged for 24 h after deposition. (e,f) High-magnification view of (c). (g,h) Hybrid film with Al electrodes; Figure S3: Transfer curve of the CsPbBr_3_/ZnO hybrid film transistor using Au electrodes; Figure S4: (a) Energy band diagram of the CsPbBr_3_/ZnO hybrid transistor. Photoresponsive characteristics of (b) the CsPbBr_3_/ZnO and (c) the ZnO transistor under 500 nm light illumination; Figure S5: Light intensity dependence of the ΔEPSC for the CsPbBr_3_/ZnO hybrid transistor; Table S1: Comparison of photoelectric synaptic devices with different structures. References [27,33,34,35,36] are cited in the Supplementary Materials.
