# Supplementary material for: Island-like Perovskite Photoelectric Synaptic Transistor with ZnO Channel Layer Deposited by Low-Temperature Atomic Layer Deposition
Source: Materials (Basel). 2025 Jun 18;18(12):2879. doi: 10.3390/ma18122879 (PMC12195490; doi:10.3390/ma18122879)
Supplement: Supplementary file 1 [file materials-18-02879-s001.zip › materials-3639940-supplementary.pdf]

materials

Supplementary Materials

Island-Like Perovskite Photoelectric Synaptic Transistor with ZnO Channel Layer Deposited by Low-Temperature Atomic Layer Deposition

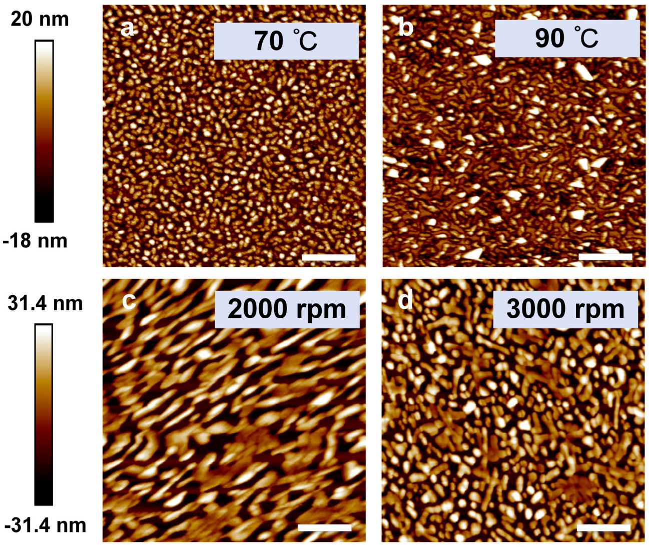

Figure S1. AFM image of $CsPbBr_3$/ZnO hybrid film with the ZnO film deposited via ALD at substrate temperature of (a) 70℃ and (b) 90℃ (scale bar: 1μm). AFM image of the $CsPbBr_3$ films spin-coated at (c) 2000 rpm and (d) 3000 rpm (scale bar: 1μm).

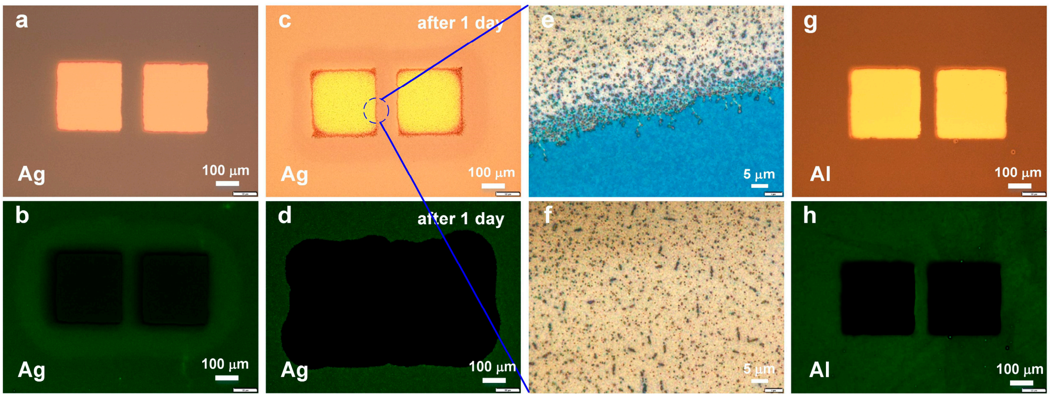

Figure S2. Microscopy images of $CsPbBr_3$/ZnO hybrid Films with silver and Al electrodes under white light (a, c, e, f, g) and blue light (b, d, h): (a, b) Hybrid film with Ag electrodes. (c, d) Hybrid film with silver electrodes aged for 24 hours after deposition. (e, f) High-magnification view of (c). (g, h) Hybrid film with Al electrodes.

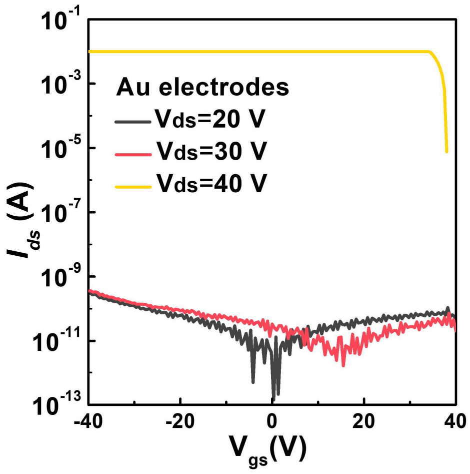

Figure S3. Transfer curve of the $CsPbBr_3$/ZnO hybrid film transistor using Au electrodes.

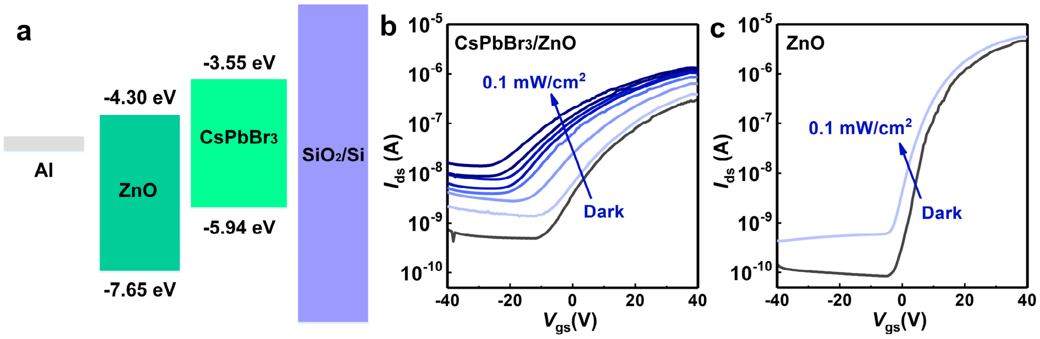

Figure S4. (a) Energy band diagram of the $CsPbBr_3$/ZnO hybrid transistor. Photoresponsive characteristics of (b) the $CsPbBr_3$/ZnO and (c) the ZnO transistor under 500 nm light illumination.

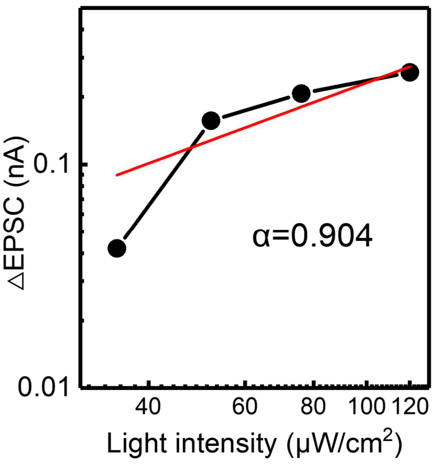

Figure S5. Light intensity dependence of the △EPSC for the $CsPbBr_3$/ZnO hybrid transistor.

Table S1. Comparison of photoelectric synaptic devices with different structures.

Device structure	Wavelength, intensity, duration of light pulses	△EPSC	Operation voltage	Quiescent current	Ref.	Year
Sputtered ZnO/$CsPbBr_3$ poly-crystalline film devices	365 nm, 0.368mW/cm^2, 1 s	~17 nA	0.5 V	~18 nA	[1]	2024
$MAPbBr_3$ single crystal /sputtered ZnO devices	520 nm, 32.5 mW/cm^2, 0.5s	~-8 nA	/	~-0.5 nA	[2]	2022
$CsPbBr_3$-MXene film/PVDT-10 phototransistors	515 nm, 0.2 mW/cm^2, 1 s	~0.25 nA	-1 V	~18 nA	[3]	2024
IGZO/Zr-AlOx phototransis-tors	405 nm, 0.4 mW/cm^2, 0.3 s	~0.12 nA	1 V	~0.18 nA	[4]	2024
ZnSe/ZnScore/shell QDs+PMMA/Petacene phototransistors	500 nm, 0.5 mW/cm^2, 2 s	~0.04 nA	/	~-0.14 nA	[5]	2024
$CsPbBr_3$ island film/ALD ZnO phototransistors	500 nm, 0.25 mW/cm^2, 0.5 s	~0.67 nA	1 V	~0.5 nA	This work	This work
Patterned $CsPbBr_3$ island film/ALD ZnO phototransistors	500 nm, 0.07 mW/cm^2, 1 s	~0.2 nA	1 V	~0.02 nA	This work	This work

References

1. Han, X.; Tao, J.; Liang, Y.; Guo, F.; Xu, Z.; Wu, W.; Tong, J.; Chen, M.; Pan, C.; Hao, J., Ultraweak light-modulated heterostructure with bidirectional photoresponse for static and dynamic image perception. *Nat. Commun.*, **2024**, *15*: 10430. https://doi.org/10.1038/s41467-024-54845-3
2. Ge, S.; Huang, F.; He, J.; Xu, Z.; Sun, Z.; Han, X.; Wang, C.; Huang, L.-B.; Pan, C., Bidirectional Photoresponse in Perovskite-ZnO Heterostructure for Fully Optical-Controlled Artificial Synapse. *Adv. Opt. Mater.*, **2022**, *10*: 2200409. https://doi.org/https://doi.org/10.1002/adom.202200409
3. Dai, Y.; Chen, G.; Huang, W.; Xu, C.; Liu, C.; Huang, Z.; Guo, T.; Chen, H., A high-linearity synaptic phototransistor based on CsPbBr3-attached MXene nanostructures for image classification and edge detection tasks. *Sci. China Mater.*, **2024**, *67*: 2246-2255. https://doi.org/10.1007/s40843-024-2965-0
4. Zhu, L.; Li, S.; Lin, J.; Zhao, Y.; Wan, X.; Sun, H.; Yan, S.; Xu, Y.; Yu, Z.; Tan, C. L.; He, G., Ultra-low power IGZO optoelectronic synaptic transistors for neuromorphic computing. *Sci. China Inf. Sci.*, **2024**, *67*: 222401. https://doi.org/10.1007/s11432-023-3966-8
5. Guo, Z.; Zhang, J.; Wang, J.; Liu, X.; Guo, P.; Sun, T.; Li, L.; Gao, H.; Xiong, L.; Huang, J., Organic Synaptic Transistors with Environmentally Friendly Core/Shell Quantum Dots for Wavelength-Selective Memory and Neuromorphic Functions. *Nano Lett.*, **2024**, *24*: 6139-6147. https://doi.org/10.1021/acs.nanolett.4c01606
